# Editorial: Tumor-associated tertiary lymphoid structures response during immunotherapy

**DOI:** 10.3389/fimmu.2025.1591820

**Published:** 2025-04-07

**Authors:** Ileana Soto Mauldin, Christine Mall Minnar, Antoine Italiano

**Affiliations:** ^1^ Department of Surgery, University of Virginia Health System, Charlottesville, VA, United States; ^2^ Center for Immuno-Oncology, Center for Cancer Research, National Cancer Institute, National Institutes of Health, Bethesda, MD, United States; ^3^ Department of Medicine, Institut Bergonié, Bordeaux, France; ^4^ Faculty of Medicine, University of Bordeaux, Bordeaux, France

**Keywords:** TLS, checkpoint blockade (ICB) therapy, immunotherapy, prognostic biomarker, ICB response

Tertiary lymphoid structures (TLSs) have emerged as critical players in tumor immunity, influencing both prognosis and response to therapy across various malignancies (1, 2). This Research Topic of *Frontiers in Immunology* brings together five significant contributions that deepen our understanding of TLSs in cancer spanning gynecological, hepatocellular, gastric malignancies, immune checkpoint inhibitor response, and high endothelial venules. Collectively, these studies illustrate the prognostic and therapeutic potential of TLSs and pave the way for future translational applications.

The review by Zhang et al. (*Front. Oncol.*) comprehensively examines the presence and prognostic significance of TLSs in gynecological cancers, including cervical, ovarian, and endometrial malignancies. The authors highlight TLSs as favorable prognostic biomarkers and discuss emerging strategies for TLS induction as a therapeutic avenue. Notably, TLS formation in these tumors correlates with improved clinical outcomes, underscoring the importance of incorporating TLS assessment into prognostic models and potentially leveraging TLSs to enhance immunotherapy effectiveness.

The systematic review and meta-analysis by Hu et al. (*Front. Immunol.*) provide robust evidence supporting the prognostic value of intratumoral TLSs in hepatocellular carcinoma (HCC). Their findings indicate that intratumoral TLSs correlate with prolonged recurrence-free survival (RFS) and reduced early recurrence, although peritumoral TLSs do not show the same prognostic value. These results suggest that TLS localization within the tumor microenvironment (TME) plays a crucial role in shaping antitumor immunity. Given the high recurrence rates in HCC, the presence of TLSs could inform patient stratification and guide personalized therapeutic approaches.

The meta-analysis by Li et al. (*Front. Immunol.*) investigates the impact of TLSs on cancer patients treated with immune checkpoint inhibitors (ICIs). The study demonstrates that high TLS levels predict improved overall survival (OS) and progression-free survival (PFS), reinforcing TLSs as promising biomarkers for immunotherapy response. Interestingly, the presence of TLSs was independent of PD-L1 expression and CD8^+^ T-cell infiltration, suggesting that TLS assessment may offer additional predictive value beyond conventional biomarkers. These findings underscore the potential for TLS-based patient selection in immunotherapy regimens.

Wang et al. (*Front. Immunol.*) present a pooled analysis on the prognostic value of tumor-associated high endothelial venules (TA-HEVs) across solid tumors. As specialized blood vessels that facilitate lymphocyte trafficking, TA-HEVs are integral to TLS formation and function. Their study highlights a significant correlation between TA-HEV positivity and improved OS, as well as disease-free survival (DFS). Given the emerging role of vascular modulation in immunotherapy, targeting TA-HEVs could represent a novel strategy to enhance TLS formation and immune cell infiltration in tumors.

The original research by Sun et al. (*Front. Immunol.*) provides a detailed analysis of TLS distribution and maturation in gastric adenocarcinoma. Their findings reveal that TLSs located at the invasive margin and those containing germinal centers are associated with superior clinical outcomes. The study also identifies dendritic cell (DC)-enriched TLSs as a key determinant of tumor immune activation. These results emphasize the need to consider both TLS location and maturation status in prognostic assessments and suggest that strategies promoting TLS maturation may enhance antitumor immunity.

The articles in this Research Topic collectively highlight the multifaceted role of TLSs in cancer. Key themes emerging from these studies include:

1. Prognostic Utility: TLSs consistently correlate with improved survival across various malignancies, reinforcing their value as prognostic biomarkers for cancer.
2. Therapeutic Potential: Strategies to induce or modulate TLS formation, including vascular targeting (TA-HEVs) and TLS maturation enhancement, hold promise for improving treatment outcomes.
3. Immunotherapy Response: TLSs may serve as predictive biomarkers for immune checkpoint blockade, offering a new layer of patient stratification beyond PD-L1 expression and T-cell infiltration.
4. Tumor-Specific Variability: TLS prognostic value varies by tumor type, location within the TME, and maturation, warranting further investigation into TLS heterogeneity and its implications for therapy.

As research advances, integrating TLS assessment into clinical practice could revolutionize cancer prognostication and immunotherapy response prediction. We anticipate that continued exploration of TLS biology will yield novel therapeutic strategies that harness the immune system’s potential to combat cancer more effectively.
